# Ausubel’s *meaningful learning* re-visited

**DOI:** 10.1007/s12144-023-04440-4

**Published:** 2023-04-26

**Authors:** T. G. K. Bryce, E. J. Blown

**Affiliations:** University of Strathclyde, Glasgow, UK

**Keywords:** Ausubel’s meaningful learning, Advance organizers, Socratic dialogue, Conceptual change, Representational and non-representational memory

## Abstract

This review provides a critique of David Ausubel’s theory of meaningful learning and the use of advance organizers in teaching. It takes into account the developments in cognition and neuroscience which have taken place in the 50 or so years since he advanced his ideas, developments which challenge our understanding of cognitive structure and the recall of prior learning. These include (i) how effective questioning to ascertain previous knowledge necessitates in-depth Socratic dialogue; (ii) how many findings in cognition and neuroscience indicate that memory may be non-representational, thereby affecting our interpretation of student recollections; (iii) the now recognised dynamism of memory; (iv) usefully regarding concepts as abilities or simulators and skills; (v) acknowledging conscious and unconscious memory and imagery; (vi) how conceptual change involves conceptual coexistence and revision; (vii) noting linguistic and neural pathways as a result of experience and neural selection; and (viii) recommending that wider concepts of scaffolding should be adopted, particularly given the increasing focus on collaborative learning in a technological world.

## Introduction

This review provides a critique of David Ausubel’s (1960, 1963, 1968, 2000) theory of *meaningful learning* and the use of *advance organizers* in teaching. We take into account developments in cognition and neuroscience which have taken place in the half century or so since he advanced his ideas, changes which challenge our understanding of cognitive structure and the recall of prior learning. We begin by evaluating the theoretical framework within which Ausubel set out his thinking. Under *Challenges concerning access to prior knowledge*, we consider how effective questioning to ascertain previous knowledge necessitates in-depth Socratic dialogue. Also, we take account of how neuroscience indicates that memory features creative, dynamic and generative processes with implications for how teachers and researchers interpret children’s responses to questions in class or during interviews.

In this context we examine the value of regarding concepts as dynamic abilities or simulators and skills rather than static constructs such as mental models. Recognizing the evidence of conscious and unconscious memory and imagery, we explore the argument that conceptual change involves selection from coexisting repertoires of knowledge rather than replacement of intuitive ideas. From the perspective of teaching and learning, we discuss the significance of linguistic and neural pathways as a result of experience and neural selection. And we recommend that individual teaching procedures such as *scaffolding* (Bruner, 1960), extension within the *Zone of Proximal Development* (ZPD) (Vygotsky, 1962), and *collaborative learning* (Day & Bryce, 2013; Johnson & Johnson, 1975) be adopted, particularly in view of the impact of COVID-19.

The paper is important because of the need for meaningful learning in an age of conflicting information via the Internet and social media replacing traditional sources of learning such as teachers, parents, significant adults, librarians and peers (see Blown & Bryce, 2020). The literature we have reviewed includes 37 which explicitly make references to *Ausubel*, *advance organizers*, *meaningful learning, subsumption theory*, or *schema theory*. Of these, 18 cover the period 2002 to 2022 with three published in 2020 and two in 2022. These include seven in the form of Power Point Presentations^1^.

Recent papers that are considered include details of advance organizers being used in teaching EFL (see Jafari & Hashim, 2012); and in professional development (see Walker, 2010; Ylvisaker, 2006), so the field remains active. We have also reviewed six articles in related fields, namely Hitzler (2022); LeCun (2022); and Marcus (2020) on Artificial Intelligence; (AI); and Demetriou, Mouyi, et al. (2022) on executive function, working memory and general intelligence “g”; Demetriou, Spanoudis, Christou, et al. (2022) on cognitive and personality predictors of school performance; and Demetriou, Spanoudis, Greiff, et al. (2022) on the development of cognitive competence and school learning.

In addition, we have taken account of the UNESCO (2022) report on the impact of AI in education worldwide.

The current review should lead to a greater understanding of Ausubel’s (1960, 1963, 1968, 2000) contribution to educational theory and greater acknowledgement of his teaching methodology – principally by evaluating his thinking in the light of results from recent neuroscience research. This indicates that memory is much more dynamic than previously thought and may be non-representational in nature. There is also evidence that early-learned ideas coexist with scientific ones with implications for teaching children how to select the most plausible answers to questions.

The study highlights the gap in knowledge between what children know and what teachers *think* individual children know and the most effective ways to close the gap.

Ascertaining what individual children actually know as a foundation for further instruction is made difficult by the move away from traditional classroom teaching. The use of social media and the Internet as sources of knowledge, together with the restrictions imposed by home learning caused by the COVID-19 pandemic, make it more important than ever that children are taught to discern between science and pseudo-science. And *advance organizers* and teaching materials must be tailored to individual children rather than whole classes. The gap in knowledge between children who are Information Technology literate and have access to Android, Smart phones, iPhones and Tablets, and computers and those who, for socio-economic or cultural reasons have no such access, is another dimension to the problem.

## A new beginning

Some 60 years ago, at a time when there was considerable interest in developmental psychology and its relevance to education, David Ausubel inspired several lines of research into school learning and instruction when he formalized the view that people learn new ideas by building on their own current knowledge. He argued that students, in seeking ways to assimilate new concepts and ways of thinking, can be helped to integrate them with what they already know, thereby bringing about *meaningful learning*. He stated, in what has become a popular maxim: “The most important single factor influencing learning is what the learner already knows. Ascertain this and teach him [sic.] accordingly.” (Ausubel, 1968, Preface, p. vi; Ausubel et al., 1978, Frontispiece.)

While certainly influenced by Piaget’s developmental theory (see Piaget, 1953), Ausubel (1963, 1968, 2000) focused on knowledge acquisition and how students, for the most part, are on the receiving end of new material when they are taught in schools - rather than discovering it for themselves, cf. Bruner (1961). Sometimes the knowledge acquired is successfully assimilated, resulting in what he termed *meaningful learning*. Sometimes the integration is weak leaving students to *rote learn* as an alternative, a term that many teachers and teacher educators use to this day, though with subtle differences in emphasis. Through his instructional research, Ausubel argued that teachers can provide *advance organizers* for new material which better ensure its meaningful learning. His definition was: “appropriately relevant and inclusive introductory materials […] introduced in advance of learning […] and presented at a higher level of abstraction, generality, and inclusiveness” (Ausubel, 1968, p. 148). Such organizers highlight what is new and important in the lesson(s) ahead; and they provide reminders of previous ideas and how they relate to what is coming. They mentally orient (or ‘set’) the learner to learn in the desired way. Not infrequently, metaphors are invoked in the course of instruction. All such strategies endeavor to actively engage the student’s mind.

Ausubel’s work underscored the important role of students’ existing knowledge, its conceptual make-up and formulation, and emphasized the *cognitive structure* in what is known by the learner. In Ausubel’s own words:

The task of teaching a subject to a child at any particular age is one of representing the structure of that subject in terms of the child’s way of viewing things. The task can be thought of as one of translation. (Ausubel, 1963, p. 30)

In *that* sense, his advice to teachers was that a student’s prior learning should be ascertained first. Prudent though that advice remains, it does indicate that – in common with many researchers of his time, and currently – Ausubel regarded memory as being *representational* in nature. The presumption was that recollection was akin to the extraction of ideas and images (most often *visual* images) from a mental database of ‘stable’ concepts (whether correct or misconceived). To quote him again: “The discriminability of a new learning task is in large measure a function of the clarity and stability of existing concepts in the learner’s cognitive structure to which it is relatable” (Ausubel, 1963, p. 89). That thinking led him to label the process of meaningful learning as *subsumption*. He recognized its significance for the individual student and therefore its idiosyncratic nature (see Ausubel, 2000). In a presentation entitled *A View on the Current Status of Ausubel’s Assimilation Theory of Learning*, published in 1993, Novak considered that Ausubel’s work had been significant in bringing about the shift in thinking about learning among psychologists – from behavioral approaches to cognitive interpretations – and to constructivism in particular^2^.

Interview techniques, both formal and informal, largely endeavor to find out how people ‘see’ things currently and assume an intactness about the ideas being described and the connections they share. It is believed that quizzing students should straightforwardly reveal what they know and don’t know. In due course we will identify the challenges which have been made to this assumption (and we will cite some of our own research on the alternative: *non-representational* memory).

## Advance organizers

Before we discuss the challenges, it is worth noting that many researchers have found Ausubel’s (1960, 1978) advance organizers helpful teaching constructs, including applications in the field of astronomy and Earth science which is of special interest to us. However they have been difficult to operationalize. We cite examples from the science education literature (including several of our own studies in children’s cosmologies). Across the educational literature as a whole, it is apparent that there are conflicting findings about the value of organizers. A possible reason has been the ‘abstract high level’ at which they have been typically pitched (*c.f.* Ausubel’s definition, mentioned earlier). Amongst the early reactions, Barron (1970) stated that: “Although Ausubel’s thinking is logically compelling, its implementation and evaluation have been beset by a number of problems” (p. 1). In his applied research with 6th to 12th grade students, he found that there was too much within-grade variation for there to be generalizable conclusions. However, he did commend the use of advance organizers by teachers to differentiate instruction with *individual* students. In a paper published 10 years later, Clark and Bean (1980) noted little support for the efficacy of advance organizers in the empirical researches they reviewed. Later still, Gurlitt et al. (2012) summarized their investigations with psychology students as follows: “With regard to education, this implies that educators should not only think about whether prior domain-specific knowledge is present, but also about how to scaffold the generation of proto-schemata at the beginning of instruction” (p. 1). The ‘scaffold’ message here is another example of representational thinking which we will return to in due course, including a critique of the construct.

Interestingly, Biser (1984) reported application of Ausubelian “principles” including advance organizers in the teaching of deaf students. And Witiw and Horton (1996) commented on the successful use of multi-modal (written, aural, visual) organizers teaching meteorology in the aviation industry. In a meta-analysis of the effects of advance organizers, Luiten et al. (1980) concluded that effects were small but that they “facilitate learning in all content areas examined, albeit broadly defined, and with individuals of all grade and ability levels” (p. 2017). Gillies (1984) reported on the use of advance organizers in surgical nursing studies: “Increased initial learning occurred with exposure to a verbal advance organizer…but intermediate and long-term retention were not significantly higher in these subjects” (p. 173). In an experimental study of students’ memories for prose material, with either concrete or abstract organizers, Corkill et al. (1988) found results for concrete forms of organizers. Citing Barnes and Clawson (1975), these authors pointed to there being clear antecedents to advance organizers dating as far back as 1900. The following year, Townsend and Clarihew‘s (1989) study investigated the effects on comprehension of verbal and pictorial advance organizers with 8-year olds, finding that only with the addition of a pictorial component did the verbal organizer facilitate understanding. According to Kumagai (2013), literature searches regarding advance organizers across three decades generated “a mixed review” (p. 3). In a recent paper (and therefore testimony to the continuing interest in Ausubel’s theory), Simöes and Voelzke (2020) describe a case study informed by an Ausubelian framework. The extent of the meaningful learning taking place through an ‘integrated technical teaching’ approach to students’ basic astronomy was assessed using comparisons between pre- and post-test data.

Other researchers have developed the field in different ways guided by Ausubel’s theory. To give four examples:

1. Novak, in his constructivist approach to learning and teaching, forcefully pursued the merits of *concept mapping*. These have been said to help students add to their knowledge, refine their understandings, and even develop their thinking in a more scientific way (Novak & Gowin, 1984; Novak & Cañas, 2008). Novak had recognized that it was not easy to counter routinely developed rote learning habits in school. He stated: “The fundamental challenge to “conceptual change teaching” is therefore to help learner’s understand how they must choose to modify their concept and propositional hierarchies and to provide instruction that is “conceptually transparent” to the learners” (Novak, 2002, p. 562).

Sharp et al. (1997) in an article describing their use of *concept maps* in interviews with 11–12 year-olds, cross-referred to Ausubel when discussing conceptual change: They commented: “It is worth remembering that conceptual change is only part of an overall learning condition (e. g. Ausubel et al., 1978…)” (Sharp et al., 1997, p. 68). The abstract and introduction themselves did show research clearly based on Ausubel’s dictum about finding out what children know and teaching them accordingly, rather than being bound by Piagetian *ages and stages*^3^. They stated:

Whilst acknowledging some of the conceptual, procedural and contextual demands imposed upon individuals as astronomical knowledge increases in complexity and conflicts with everyday experience, many involved in the primary sector at all levels who have mastered some degree of subject and pedagogical knowledge in astronomy agree that it can be made readily accessible to children in the primary years provided it is presented to them in a developmentally appropriate way and at a developmentally appropriate time (pp. 67–68).

Nussbaum and Novak (1976) were more explicit about the value of applying Ausubel’s theory in their study of children’s concepts of Earth. (We will return to ‘conceptual change’ in detail later.)

2. Rumelhart and Ortony’s (1977) work on *schema theory*^4^ analyzed memory and what was then known about how the knowledge in students’ prior learning and memory should be considered. Their argument was supportive of Ausubel’s emphasis on higher-order structuring in advance organizers. Notably however, in a later paper, memory representation was judged to be “far richer and detailed than schema theory would suggest” (Alba & Hasher, 1983, p. 283).
3. Cuevas (2012) combined scaffolding with schema theory in an experimental study of reading comprehension among urban high school students. He asked participants in a treatment group to paraphrase statements they read as organizers “to assist with the encoding of information into long term memory” (p. 29). His conclusion recommended that: “Treatments of this sort could be incorporated into standard curricula and possibly help produce widespread gains in student reading comprehension” (p. 35). What we report below about research on non-representational memory has much more to say on matters relating to meaningful learning and signal complications regarding advance organizers and the original hope for their use in teaching.
4. Another influence of Ausubelian thinking is apparent in work on *deep learning.*

In this branch of educational research, the approaches to learning by students in higher education have focused on their intentions when starting, then attempting to progress through learning tasks, memorizing, revisions, assessment, etc. To quote from the review by Baeton et al. (2010), research efforts have looked at “how to optimize students’ approaches to learning towards deep, meaningful learning by means of implementing student-centred teaching methods…” (p. 244). Early researchers often cited in this field include Biggs (2001), Entwistle (1998); Marton and Säljö (1997). Entwistle noted that “where [they] have acquired extensive prior knowledge and understanding, most students assimilate the material quite readily by what has been called *meaningful reception learning* (Ausubel et al., 1978)” (Entwistle, 1998, p. 9). Baeton et al. (2010) observed that “where teachers are involved and oriented towards students and changing their conceptions, students are inclined to use a deep approach” (Abstract, p. 1).

## Challenges concerning access to prior knowledge

In the rest of this paper, we seek to re-appraise Ausubel’s (1963, 1968, 2000) thinking in the light of more recent research on inter-related complexities in cognition and instructional psychology. We will argue for subtler interpretations of how teachers should regard the thinking processes which learners might be using as they are helped to meaningfully learn. Thus we offer some new insights into how instruction utilizing scientific ways of thinking can be progressed. First, it is appropriate to comment on the obvious way in which teachers and researchers try to gain access to students’ thinking: they ask questions.

### Questioning in classrooms and online

Advice about classroom questioning has figured prominently in the educational literature for some time, much of it targeted at pre-service teachers. Despite the wide scope of the text by Ausubel et al. (1978), there was surprisingly little discussion of questioning. Indeed the index to the book does not include *questions, questioning, classroom interaction*, or *dialogue (Socratic* or otherwise^5^). *Chapter 1*3, devoted to ‘Group and Social Factors in Learning’ retains the individual focus on issues, but without any discussion of whether or how teachers might question students, or to what end. However the authors did state in that chapter:

“Individualized instruction is much more effective than instruction in groups, except in learning situations where the material is more controversial and learners require cross-fertilisation and exposure to other views” (p. 462). The concession here receives no elaboration. It would be fair to say that such matters were not considered central to the debate at that time (something we will return to later) but, overall, it can be said that Ausubel significantly underplayed a key part of what takes place in classrooms. His discussion of how students’ prior knowledge should be conceptualized omits how it may be ascertained.

[In our own area of interest – children’s cosmologies – we are aware of Agan and Sneider (2004) using a one page questionnaire on Earth shape as an assessment instrument to ascertain what individual children knew before instruction but it assumed a spherical Earth in its accompanying illustrations and would not have invited intuitive or synthetic cosmologies based on non-spherical models of the Earth.]

In general and for some time, most educational texts focus upon strategies that will help individual students and their immediate peers to think productively and shape the lesson in ways suited to the teacher’s objectives. For example, in the UK, Cohen et al. (2004) 5th edition text, was typical in embedding recommendations about questions in its section on *language in classrooms* – purposes served by different types, prompting, probing, take-up, and so forth. Black and Wiliam’s (2006) book focused upon the role of questions as part of ongoing formative assessment in the classroom. Fusco (2013) considered that classroom discussion encouraged young learners to become reflective, responsible thinkers. Her practical advice about questioning and Socratic dialogue is useful in contexts where there is often resistance to spending time on discussion in the face of pressures from standardized testing. Also addressing US teachers, Francis (2016) focused on ‘promoting cognitive rigor’ through well-designed questioning techniques.

Answering questions in science is of course influenced by how students are taught, their experience of receptive take-up by teachers, and how lessons are conducted (whether ‘practical’ or ‘theory’). As part of a study looking at the development of high-school chemistry students’ ability to ask more and better questions, Hofstein et al. (2005) found that those students who had experience in asking questions in an ‘enquiry-oriented teaching lab’ (practical chemistry class), performed better than students taught more traditionally.

For all subjects and levels of education, there is also a plethora of Internet websites, many of them providing ‘tips for teachers’ about what to do and what not to do for whole-class, small-group and individual questioning. What is common to all of them is a concern for the teacher to uncover what students ‘know’; to find ‘aspects’ of their knowledge; to discover ‘information’ they have to hand; to become aware of ‘partial understandings’, or ’shades’ of their comprehension; and so forth. The advice is to find out what a pupil, or a group of pupils, or the whole class, see or grasp at the start of a lesson; what is in their minds *vis-à-vis* what is to come. Low level (closed or convergent) questioning is often criticized in favor of high level (open or divergent) questioning, and it is generally recognized that one-word answers limit class dialogue and the involvement of more pupils - see, for example, Doherty’s (2021) posting on *Skilful questioning: The beating heart of good pedagogy*. Citing several researchers over recent years, Doherty stated that:

Simply asking higher-cognitive questions does not necessarily produce higher-cognitive responses from students… On balance, low-level questioning aimed at recall and fundamental-level comprehension will plateau classroom learning quickly. Higher-level questions can produce deeper learning and thinking, but a balance needs to be struck. Both have a place and a mixture of questions is recommended.

Heick’s (2021) posting on *Why questions are more important than answers* included encouragement to teachers to constantly practice their strategies to better ensure that students reveal their thinking:

When you ask questions – in exams, in person, in your next Socratic discussion– insist on good questions. Great questions. Model their development. Revise their wording. Toy with their tone. Simplify their syntax or implications over and over again until the confusion has been bleached and there’s only thinking left.

(Heick’s use of ‘Socratic discussion’ in this context seems somewhat inadequate, as will become apparent from our later discussion.)

From a research perspective, it is evident that all of the advice in textbooks and on websites is based on a *representational* notion of (student) memory. Before we address this, it will be helpful to consider the matter of *interviewing* intended to find out what children know – both the informal kind in daily use by teachers and the formal varieties which researchers draw on. Questioning in the classroom and online requires skill on the part of parents and teachers to develop and enhance children’s ability to discriminate between true and false information. The reality in the developed world at least, is that children are becoming more responsible than ever for their own education bringing new meaning to the term “self-taught”. Ausubel would have wished that such learning was “meaningful” and not based on pseudo-science or misinformation. Skill in discerning one from the other should be a priority objective of education.

### The interview as an inter view

As stated above, ‘interviewing’ can be viewed informally, as part-and-parcel of teachers’ everyday interactions with students during lessons, or more formally where teachers are involved in research. Whichever, they need to be able to probe deeply into children’s thinking – either as they quiz pupils and discuss their answers, or react to semi-structured interview questions which are often set out in advance in an interview guide. Kvale and Brinkmann (2009) stressed that an interview is an *inter view*, where “knowledge is constructed in the inter-action between the interviewer *and* the interviewee” (p. viii). To conduct sensitive questioning during research, subjects’ responses need to be considered cautiously. Arguably, this confers a useful advantage to those with experience of teaching (here science teaching). It requires: (a) robust, relevant knowledge of the subject matter, and (b) the ability to handle Socratic dialogue to best advantage. In a sense, interview guide questions are essentially static (to give stability and validity, especially to longitudinal developmental studies with repeated measures). Whereas Socratic dialogue and scaffolding are dynamic processes resulting spontaneously from guide questions, and used by the experienced interviewer with appropriate *content knowledge* and *pedagogical content knowledge*^6^.

Socratic dialogue is much more dynamic and interactive than pre-planned questions in the sense hinted at by Heick (2021) referred to in the previous section. The dialogue must be guided by children’s responses and it is worth remembering that Piaget founded his clinical method on flexible questioning rather than following a script or interview guide. He had criticized ‘tests’ in his 1930 text because the method “does not allow a sufficient analysis of the results” and it “falsifies the natural mental inclination of the subject or at least risks doing so” (Piaget, 1930, p. 3). He went on to say that: “The only way to avoid such difficulties is to vary the questions, to make counter-suggestions, in short, to give up all idea of a fixed questionnaire” (Piaget, 1930, p. 4). This one-to-one open-ended questioning technique was what came to be known as the *clinical method*. We have justified our use of an *interview guide* (Blown & Bryce, 2020, Appendix A) to provide continuity and validity in our own longitudinal research studies and thus ensure that the same key questions are asked in the same context in repeated interviews^7^. However we have diverged into Socratic dialogue whenever it was judged to be appropriate (see Blown & Bryce, 2013, 2017, 2020, 2022; Bryce & Blown, 2016, 2021).

As for all forms of questioning, checking responses must be carried out carefully to avoid ‘foreclosure’ on judgments about young people’s understandings. Furthermore, and from the instructional perspective, teachers need to be able to probe deeply into students’ thinking when they cross-question them, and teach by scaffolding as the opportunities arise. Consistent with Ausubelian principles, they must be mindful of when they are engaging in Socratic dialogue, and with what consequence in how they judge students’ understandings. This can be far from easy.

Dialogue involving Socratic questioning has a crucial role in determining the interviewee’s intended meanings, and thus why the researcher’s own content knowledge plays a vital role. Sometimes there are circumstances where opportunities to fully exploit Socratic dialogue may not arise, in which case a second-best strategy may have to be used. In Blown and Bryce (2017) we identified teachers, librarians and parents as primary sources of young people’s astronomical knowledge. We have reported cases where children were referred to class teachers and librarians for further information to address queries arising from interviews. In such cases, this was to avoid breaking with Socratic tradition where we try to ascertain what the child already knows, arguably more in keeping with Vygotsky’s (2012) and Bruner’s (1960) construct of scaffolding than with the original Ausubelian principles. Typically, the child views the researcher as a teacher and expects to be taught. Pedagogical conflict arises because the *interviewer as researcher* endeavors to maintain a degree of objectivity and avoid imparting knowledge; whereas the *interviewer as teacher* naturally wishes to be attentive to the individual student and teach. On this score, any re-appraisal of Ausubel’s (1960, 1963, 1968, 1978, 2000) work should usefully consider *both* how teachers instruct students and how interviewers cross-examine their subjects. In a recent article reviewing all the historical and recently published articles on children’s basic astronomy knowledge, we have provided a critique of the methodological difficulties that have arisen with many of the interview strategies developed by successive teams of researchers (Blown & Bryce, 2022).

### Representational versus non-representational interpretations of memory

Conventional thinking has considered the act of remembering to be a *reproductive* or *replicative* act. Verbalized meanings and visual images which constitute knowledge are thought to be re-activated and brought into play during interactions, typically in response to questions asked by a teacher or peer, or interviewer. The notion of *representation*, with its implication of symbolic activity, traditionally suggests that people are repeating or re-visioning what they were taught (or found out) on a previous occasion when reacting to questions. Any act of recollection certainly has significance for individuals personally: consciousness tells us that *that* is how it is. In other words, what comes with any recall is a built-in degree of confidence that what we are remembering now is what we learned and have recalled previously. Not because of any exaggerated pride in certainty, or super-conscious reflexivity, but simply that human cognition processes work in this way. It is what the neuroscientist Edelman (1989, 2001) called ‘consciousness and the remembered present’ when he argued about what was taking place – and argued very differently from tradition concerning *non-representational* memory. The complex interconnections of neural groups in the brain ensure that there is more dynamism to the processes of recollection than a simple reactivation. Edelman’s interpretation was that remembering involves a *creative* action, conferred by the cognitive efficiency of the human brain (which, subjectively, we all feel we ‘know’ so well). Frequently, more is triggered in reaction to a question and can result in a number of different responses by the same person, at different times, to what was sought by the question. Ideally of course, this can be in accord with Ausubel’s (1960, 1963) meaningful learning, but very many variants, partial understandings or misconstructions are possible. Also, people can change modes as they begin to recollect things, e.g. switching from verbal recall to imagery. Edelman and Changeux (2001) stated:

A dynamic nonrepresentational memory … has properties that allow perception to alter recall, and recall to alter perception. It has no fixed capacity limit since it actually generates ‘information’ by construction…It is robust, dynamic, associative and adaptive. If such a view is correct, every act of perception is to some degree an act of creation and every act of memory is to some degree an act of the imagination. Biological memory is creative and not strictly replicative. (p. 56)

One of our own investigations (Bryce & Blown, 2021) looked at children’s memory of the configurations and shapes of the Earth, the Sun and the Moon over short then longer/later intervals of time. It was apparent that recollections were not identical. Basic expressions or patterns were often similar but many children who did not report images during any interview, proceeded to conceptualize effectively – that is, explain or draw or model their thinking – at later times.

Novak (1993), referred to in our introduction and published at a time when memory was firmly regarded as representational, discussed (see p. 10) how the “Integrative reconciliation of concepts and propositions in cognitive structure is required for elimination of misconceptions” (or ‘alternative conceptions’, see below). In a detailed article, also published in 1993, Smith et al. (1993) provided a careful analysis of the thinking of that era regarding children’s *misconceptions* and how focused instruction had to ensure their replacement by scientifically correct ideas. They argued that the discontinuity between student learners’ ideas and those of experts was over emphasized, indeed that it was in conflict with constructivist thinking. While they did not cite Ausubel among their references, they did acknowledge his work in referring to “the basic premise of constructivism: that students build more advanced knowledge from prior understandings” (p. 115). From about that time, the term ‘alternative framework’ became commonly used in preference to ‘misconception’ in the educational research literature, much to the credit of Rosalind Driver (Driver et al. (Eds), 1985). See also Driver and Easley (1978); Driver (1981); Gilbert et al. (1982); Nussbaum and Novick (1982); Osborne and Freyberg (1985); Kyle and Shymansky (1989); and Bryce and Blown (2012). Later, we will show that by regarding concepts as *coexisting*, rather than being serially replaced in the course of instruction and development, “incorrect ideas” are more often than not “ideas” being *selected* from an everyday rather than a scientific repertoire of expressions founded on a scientific paradigm or worldview.

In parallel with Edelman’s (1987) work *Neural Darwinism: The theory of neuronal group selection* which argued in favor of memory being non-representational, there have been alternative theories based on neuroscience that the brain features neural representations.

Clark (2013) detailed a model of the brain in which neuronal networks are dedicated to minimizing the error between predictions about the world based on past experience and actual current information based on the senses. He summarized possible forms of neural representation as follows:

Neural representations, should the hierarchical predictive processing account prove correct, encode probability density distributions in the form of a probabilistic generative model, … a departure from traditional understandings of internal representation, and one whose full implications have yet to be understood. It means that the nervous system is fundamentally adapted to deal with uncertainty, noise, and ambiguity, and that it requires some (perhaps several) concrete means of internally representing uncertainty.

The advances in neuroscience which informed the work of Edelman et al. have resulted in a re-assessment of memory representation from the traditional static filing system to a much more dynamic form featuring groups of neurons that compare old information with new concepts and percepts to create a constantly updated mental model of the world. Such a model of cognition and perception creating memory has implications not only for learning and teaching in traditional psychology but also for machine learning and AI as pioneered by Turing. Although the full implications of Edelman’s (1987) theory of neural group selection with nonrepresentational memory and Clark’s (2013) theory of hierarchical predictive processing with internal representation of reality are yet to develop from neuroscience and other areas of psychological research; four themes of interest emerge:

- In AI: Hitzler (2022); LeCun (2022); and Marcus (2020) indicate the way ahead.
- In the Perception/Cognition interface: Goldstone and Barsalou (1998); Nanay (2013); Smith et al. (2013); and Tacca (2011) argue the case for perceptual representations and the merging of previously independent fields of psychological enquiry.
- In neuroscience: Ahissar and Assa (2016); Clark (2013); deCharms and Zador (2000); report details of neuronal representations in perception, cognition and memory.
- And, of special interest to the current authors: Williams (2019) highlights the perception/cognition interface, mental representation and domain specific intuitive theories.

### The dynamism of memory: an analogy for non-representational memory

In Bryce and Blown (2016, 2021) we set out in detail much of the recent thinking by neuroscientists and cognitive psychologists concerning the working of human memory and applied their arguments to our interview research on children’s cosmologies. We emphasized Edelman’s work on the dynamism of memory; in particular *re-entry processes* which result in near-instantaneous links (words, images, etc.) being made at the point of recall. In arguing that the brain is *selectional*, Edelman (2006) stated: “memory, imaging, and thought itself all depend on the brain ‘speaking to itself’ by re-entry” (p. 57); “memory [is] a dynamic recategorical system property” (p. 59). This is consistent with neuroscientists like Kiefer and Pulvermuller (2011) who wrote about concepts being *flexible*: “conceptual flexibility implies that access to a concept during language comprehension or thinking cannot be conceived as a replay of stored sensory-motor information as in a movie, but as a context-specific situation-dependent dynamic activation process” (p. 809). We say more about the term ‘concept’ below.

Prior to his work in neuroscience and in philosophy of mind, Edelman’s Nobel Prizewinning research concerned the structure of antibody molecules. Edelman and Tononi (2001) made an interesting comparison between conscious memory, how it enables us to be cognitively well organized, and how antigens enable our immune system to be biologically effective:

An antibody is not a representation of a foreign antigen, yet through the system of immunological memory it and other antibodies can recognize that antigen. An animal can be well adapted to an environment but is not a representation of that environment. Similarly, a memory is not a representation; it is a reflection of how the brain has changed its dynamics in a way that allows the repetition of a performance. (pp. 94–95)

Hence, with regard to the act of remembering, an individual is actively formulating images and explanations in an intersected way during the processes of recollection, drawing on available thoughts. If persuaded by Edelman and Tononi’s analogy, the quotation indicates that “performance” should be interpreted as in a dance or acting or playing music. No two performances are identical even although the same routine or script or score is used: the process of creation results in slightly different outputs; sometimes very different creatively as in jazz – as we have found with children’s verbal responses, drawings and models. Irrespective of audience, a self-explanation is significant to the immediacy of what is depicted or framed now, and to future acts of recall. He or she is re-generating them each time and in ways which, thereafter, subsequently alter remembered images and explanations in the area concerned. More recently, researchers like Edelman, and others, refer to the *dynamic core* of the brain generated by the re-entry processes which link up dispersed areas of the cortex and account for the relation between perception and conscious memory (see Edelman et al., 2011). Recall is re-enactment of neural pathways to produce what seems like a memory but it is not akin to a file or recording or material thing. Scaruffi (2000), who has been critical of the writing of Edelman and Tononi, did however align himself with their view of the non-representational nature of memory as follows:

A memory is a creative reconstruction of the neural activity needed to repeat an action. Memory is “constructive re-categorization”. There are thousands of memory systems in the brain: memory is not a brain region but a property of brain regions. Memory is the process by which brain regions collaborate to produce an output similar to a previous output. In general, the neural activity related to the same “memory” will be different at different times. If a few neurons die, the memory of an event might survive precisely because there are ways to reconstruct it that don’t depend on the existence of those specific neurons. This makes memory more robust than a simple representation of events. Its capacity is also bigger than the size of the brain, just like a computer program can generate many more statements than the statements that constitute the program itself. Memory is creative, not replicative. (Scaruffi, p. 3)

Once again, Ausubelian thinking about prior knowledge needs to be considered carefully. Teachers need to explore students’ responses with caution; encouraging them to check their thinking multi-modally (as we concluded the implications of our report in Bryce & Blown, 2016). Immediate responses in any dialogue with a learner are typically ‘the tip of the iceberg’.

Our studies of children’s cosmologies of the Earth, Sun and Moon revealed dynamic switching between and within everyday and scientific paradigms of knowledge manifest as coherent world views and their associated repertoires of concepts in the form of abilities and skills (see Barsalou, 2003 below). Our multi-modal methodology utilizing verbal language, drawing and play-dough modeling, complemented by Socratic dialogue, enabled children to share their cosmologies with the researcher in depth. There was evidence that information in the symbolic form of categories of cosmological concepts such as Earth shape was being evaluated rapidly multi-modally and interactively with the researcher in real time to select the most plausible and suitable response to questions (see Blown & Bryce, 2010, 2017, 2022; Bryce & Blown, 2016, 2021).

### Concepts as abilities or simulators and skills

To put the argument slightly differently, the standard interpretation of the recall process is that “it” results in the revelation of a concept or misconception because ‘it’ is there, possibly amongst others, to be found. According to some theorists (e.g. Barsalou, 2003; Barsalou et al., 2003), the very notion of a *concept* should be dispensed with if one views matters non-representationally. Rather, concepts should be viewed as *abilities* or *skills*. This somewhat counter-intuitive idea stems from recognizing that the afore-mentioned dynamism of memory means that an attempt to remember something begins with the brain searching, in some *automatic* and *unconscious* way, for relevant links in (or via) multiple ‘modes’, i.e. verbal expressions or associations and visual images. It does so because of the interconnectivity of so many areas of the brain. In Edelman’s words, successful memory results “from the selective matching that occurs between ongoing, distributed neural activity and various signals coming from the world, the body, and the brain itself” (Edelman, 2000, p. 93). And, “consciousness arises as a result of integration of many inputs by re-entrant interactions in the dynamic core” (Edelman, 2003, p. 5524).

At the risk of oversimplifying things, there may be a final verbalized (or drawn or modeled) ‘thought’ at the stage of articulating a response, but it results from memory re-categorizing earlier associations – ‘live’, so-to-speak, not by finding pre-prepared representations. Researchers should *not* be thinking of knowledge in an *amodal* sense: Edelman’s argument is that knowledge is grounded in modality-specific systems. That is what makes our minds so powerful. Barsalou, writing from the representational viewpoint, states that: “According to the situated action view, a concept is not a general description used over and over again across situations. Instead a concept is an ability or skill to construct specific representations that support different courses of situated action” (Barsalou, 2003, pp. 545/546)^8^.

These deliberations run counter to Ausubel’s (1960, 1963, 1968, 2000) original thinking that conceptual knowledge was highly structured, representational and non-dynamic in nature. Researchers and teachers now need to concede the dynamism of cognition, to think differently about ‘structure’ and acknowledge, in a very deep sense, that exchanging ideas with another person (as in question and answer sessions in the classroom or during an interview) requires very sophisticated Socratic dialogue to reach any surety about what they have in their mind.

### Conscious and unconscious memory and imagery

Each time an internal or external cue triggers a conscious reflection on present or past events, memories are regenerated – hence the constant updating which our minds are subject to as individuals exchange ideas with other people. The cues can be personal musings, questions asked by teachers during a lesson, thoughts stimulated as part of Socratic dialogue, and so forth. The conscious reflections, including inner speech (Vygotsky, 2012), very often involve imagery, though according to the neuroscientist Dresp-Langley (2012), an internal cue may even result from unconscious associations. Thus imagery and memory are features of consciousness itself. However, as Edelman argued: “We experience primary consciousness as a ‘picture’ or a ‘mental image’ of ongoing categorized events. But…there is no actual image or sketch in the brain. The ‘image’ is a correlation between different kinds of categorizations” (Edelman, 2001, p. 119). See also Edelman (2005).

Ausubel’s discussion of meaning and ‘representation’ which he used in relation to successful verbal learning had a slightly different focus. He wrote: “Meaning, therefore, in our view, always implies some form of representational equivalence between language (or symbols) and mental content” (Ausubel, 1963, p. 35). Had Ausubel been aware of the recent cognitive and neuro-scientific research to which researchers are now privileged, especially the possible non-representational nature of memory, he would not have been so assured of the structure he assumed was a feature of prior learning. The evidence that we have adduced (see Bryce & Blown, 2021) shows that imagination has both conscious (representational) and non-conscious (non-representational) forms. As we stated in that article, there will be interactivity or influences between images and explanations as responses are formulated by any respondent to questioning, influences which are largely unconscious and possibly unknowable to the outsider. These are in accord with the results of Hoenig et al. (2008, p. 18): “conceptual features contribute to a concept to varying degrees in a flexible context-dependent manner”. We anticipate that further brain research should clarify the relationship between conscious and unconscious mental processes.

### Conceptual change versus conceptual coexistence, selection and inhibition

It has always been assumed (and hoped) that, with development and schooling, children’s early-learned ideas will be revised; more scientific concepts will be acquired, and steadily used instead. For researchers in science education, the concern for many decades has therefore been about how to conceptualize *conceptual change.* Vosniadou (2013) gave an overview to this topic and von Aufschnaiter and Rogge (2014) provided a comprehensive review of it, pointing out that: “Conceptual change is part of learning but not all learning is conceptual change” (p. 1). They credited Ausubel for stimulating research into the exact relationship between existing cognitions and ones which are being newly acquired, and stated that: “Predominantly, research on conceptual change is based on a constructivist epistemology assuming that concepts are a result of personal or social constructions” (p. 1).

Piaget’s (1970) *accommodation* mechanism had recognized that newly acquired schemas did not take the place of earlier conceptions; multiple explanatory notions could co-exist. However, whereas Piaget’s theory of ages and stages constrained development in all domains (see Donaldson, 1978), researcher thinking of the 1980s considered that changes in cognitive structures would be *domain-specific.* These could take weak or strong forms (see Posner et al., 1982; Carey, 1985; Driver & Easley, 1978; Vosniadou & Brewer, 1987; and others). Vosniadou and Brewer (1992, 1994) used the term ‘synthetic models’ to indicate how children’s ideas incorporate elements of everyday and scientific concepts. New knowledge is being evaluated for relevance when it is linked into and constrained by underlying cognitive structures.

Hypothesized *concept maps* should be unique for each individual, being an amalgam of life experience; cultural influences from parents and grandparents; and scientific ideas from teachers at school. Paradoxically, although researchers and teachers wish every child to ‘know’ the scientific view, they also wish them to retain their individuality since that is the source of creativity. In a way, the very existence of individual differences despite schooling indicates that whatever is going on in memory involves dynamic processes of selection from a variety of repertoires.

Our own research (Bryce & Blown, 2006; Blown & Bryce, 2017) has certainly revealed children’s ideas changing with age and education. These included cases where children were able to visualize their concepts and manipulate them dynamically by a rapid switching of concepts during an interview. However, as for all researchers, the nature of the processes involved in conceptual change were, and are, elusive. For example, one has to interpret triangulated data – verbal statements/drawings/models – without being able to see directly the conceptual images that children are attempting to share.

As we have explored in detail in Bryce and Blown (2021), research thinking from about the mid-1980s onwards firmly acknowledged the *coexistence* of everyday (or ‘naïve’) ideas and scientific concepts. Empirical findings accrued to emphasize that several manifestations of an idea continue to exist during any verbal exchange. What is brought to mind, or constructed on the spot in an interview or lesson, is dependent on the context of the questioning (see Driver et al., 1994; Duit, 1994; Siegal et al., 2004; Vosniadou, 2014; Potvin, 2017; and ourselves, Bryce & Blown, 2016, 2021; Blown and Bryce, 2017). For example, Nadelson et al. (2018) stated:

… we support the position of Ohlsson (2009) and maintain that rather than going through a process of restructuring conceptions, learners instead adopt and form the new conceptions as their dominant conception to explain phenomenon while effectively maintaining prior conceptions in a dormant or suppressed state. (p.155)

These writers provided a comprehensive model (what they refer to as a Dynamic Model of Conceptual Change, DMCC) embracing the many variables and contextualizing facilitations or constraints surrounding conceptual change.

Research findings from neuroscience have corroborated the ideas of psychologists and educational researchers. For example, Brault Foisy et al. (2015) considered that coexisting images or memories or concepts are being compared during delays which subjects make in their responses to questions put to them during experiments^9^. Furthermore, other evidence from neuroscience suggests that initial concepts are *suppressed* by a process of *inhibition* – but continue to function as alternative repertoires in memory (see Mareschal, 2016).

Once again, the importance of Socratic dialogue is apparent in efforts to understand the knowledge which children possess in a particular domain. An ‘Ausubel of the present decade’ might argue that one still needs to “ascertain …what the learner already knows”, though we can so easily underestimate that knowledge, if care is not taken and in-depth questioning is not pursued. Some researchers would now argue that rather than a focus on “conceptual change”, suppositions should concentrate on “concept selection”. The processes are more about “conceptual re-prioritizing” or “reassessment” rather than “restructuring”. Being subject to the same revisions as dynamic memory, the contents of a repertoire of ideas may change but the repertoire as a category remains unchanged; i.e., everyday, cultural, scientific vocabularies or language modes. What is changing in the process of “conceptual change” is not the concepts as such or the repertoires to which they belong but rather the selection of concepts or skills that best fit the context or situation. Concept acquisition probably involves some form of sorting into categories or repertoires; these being the bases from which conceptual selections are made in response to interview questions. Memory and repertoires are in a state of flux, but it is controlled flux, with the brain maintaining “our feet on the ground” to make sense of new information about the world and relate it to older ideas.

An analogy may help here, following Barsalou’s (2003) argument that concepts are creative and re-creative *skills* involving the ability to create the essence of a situation repeatedly in memory and apply it multi-modally (in speech, writing, drawing, modeling). Conceptual selection enables the child to select the most suitable concept (select the most suitable tool and apply the most appropriate skill) from a range or repertoire of alternatives (everyday, cultural, scientific) to solve the immediate situated problem. Conceptual-coexistence is analogous to the range of tools in a skilled tradesman’s apron or toolkit (some old, some new, none discarded). Conceptual change is analogous to the tradesman switching tools or creating a new tool while resolving a problem and fixing it. Critically, the old tool is kept, and the new one added, the range of options available for selection changes, and the skill of the craftsman increases, but nothing is lost or replaced in the apron or toolkit. New skills are added and repertoires are enhanced.

To return to Ausubel and educational practice, successful classroom strategies pivot on the teacher’s ingenuity in devising advance organizers and mental sets likely to bring about understanding. These sets should enable the learner to access the best of their knowledge in the circumstances, so the teacher needs to be receptive to alternative repertoires being used by learners. Sensitive teaching generates a transparently open *reciprocity* of understandings; learners should appreciate what their teacher is trying to achieve; he/she must work with their endeavors; both parties should know what they are mutually concerned to do during instruction.

### Linguistic and neural pathways as a result of experience and neural selection

Edelman described how the brain’s cognitive functions work together, or near simultaneously (see Edelman, 1987, for a discussion of *Neural Darwinism: The Theory of Neuronal Group Selection*; how selective processes along Darwinian lines favor some neuronal groups over others leading to more efficient cognition). He considered that re-entry processes (resulting from the massive inter-connectivity of neural *networks*) create concepts: “concepts are the outcome of the brain mapping its own perceptual maps leading to generalities or ‘universals’. While memory and concepts are, together with value systems, necessary for meaning or semantic content, they are not identical to that content” (Edelman, 2005, p. 104).

See Smoliar (1989) for a book review of *Neural Darwinism* published in *Artificial Intelligence* (see recent references to AI below).

Interestingly, Ausubel’s discussions of meaningful learning assumed the existence of structures incorporating conceptual *traces*, these being organized hierarchically (see pp. 24–29 of Ausubel, 1963). He explicitly refrained from considering terms in neurophysiology about which very little was known at that time. In a footnote, he wrote:

The term “trace” is used here simply as a hypothetical construct to account for the continuing representation of past experience in the nervous system and in present cognitive structure. No assumptions are made regarding the neurophysiological basis of the trace or regarding psychophysiological correlations (Ausubel, 1963, p. 24).

With hindsight, he would be intrigued by present-day neuroscientists’ investigations on the brain’s circuitry, in particular on the similarity between Edelman’s *neural pathways* and his own *traces*. Researchers and teachers can only guess as to what he would have made of the *dynamism of memory* which is a consequence of their interconnectedness.

A different perspective arises from the work of psycholinguists who have long noted the developmental changes taking place in young children by virtue of their experience of language ‘alone’, i.e. “Experience of particular language forms may be all that is required for internal organizational processes to operate” (Cromer, 1987, p. 223). Cromer’s point was made on the basis of his longitudinal research with children. He noted changes taking place independently of any feedback from researchers or teachers concerning the correctness of any of their responses to questions put to them at different survey stages. He considered that exposure to a linguistic structure in itself induces the child to operate on that structure, leading to a *reorganization* of linguistic knowledge. Cromer’s ‘linguistic pathways’, as well as Edelman’s ‘neural pathways’, have echoes of Ausubel’s ‘traces’.

### Scaffolding in context and the increase in collaborative learning

In our article about children switching between everyday and scientific language – in both directions (Blown & Bryce, 2017) – we reviewed the literature concerned with classroom language and efforts to scaffold students’ learning. Scaffolding can reinstate everyday, intuitive ideas as the foundation of later scientific learning. We supported Lemke’s (1990) recommendation that: “Teachers should express all semantic relations among terms, and all conceptual relationships for each topic, in ordinary colloquial language as well as in scientific language, insofar as possible, and clearly signal when they are using each” (pp. 172/173).

Notably, through several editions of *Thought and Language*, Vygotsky (2012) reasoned that everyday, spontaneous concepts and scientific concepts were in continual interaction. For him, the ZPD was the meeting place of everyday and scientific concepts. In practice, the science teacher has the complications (as well as the advantages) of working with students in groups in laboratories. From moment to moment, the immediate and very localized topic of conversation varies. Individuals hear and share (or don’t share) explanations, either with each other, or with the teacher. Even in well-managed circumstances with an instructor well disposed to Ausubelian principles, the flow between colloquial and scientific expressions among groups of students can be a challenge. A successful exchange between peers can, a few minutes later, have useful scaffolding undone due to an aside from another individual beyond the teacher’s hearing. If a misconception is seeded, it becomes difficult to supplant it in a pupil’s mind with better conceptual understanding. Hence the value of Socratic dialogue to encourage students to critically examine their ideas on a topic.

A further complication arises from efforts to deliberately bring about *collaborative learning*; that is where students are trained to work together to maximize their own and each other’s learning through discussion: see Day and Bryce (2013) for a review and empirical trials of its introduction and management in secondary schools. Collaborative learning (following the inspirational work of Johnson & Johnson, 1975) encourages pupils to work together in small, heterogeneous groups to produce a group product, or arrive at an agreed-upon decision. The pupils know that they cannot reach their learning goals if any of the others in the group do not complete their tasks. The work need not involve practical lab work; indeed the pedagogical strategy has become prominent in the handling of socio-scientific, controversial issues like global warming, genetic modification, cloning, vaccines, and so forth (see Bryce and Day, 2014a, b). Vigilance is required on the teacher’s part during such discussions to regularly monitor and challenge pupils’ thinking.

Returning to Ausubel, we quoted him in the introduction as having stated that the teaching of scientific concepts using everyday ideas could be thought of as a ‘translation’ of the child’s way of thinking to the accepted scientific wisdom. In the light of recent research, it would be better to think of giving the young person *both* repertoires and the *skill to discriminate* between them so that he/she can in future *select* the most appropriate concepts for the situation to hand. Researchers and teachers need not seek to *replace* early learned cultural ideas with school learned scientific concepts; they can aim towards conceptual precision in both everyday and scientific language, with an emphasis on conceptual equivalence (e.g., in children’s cosmologies research, “Ground” being equivalent to “Earth” in younger children).

Bruner’s (1960) engineering metaphor of scaffolding has been used to describe how instruction can build scientific concepts on everyday ones through *bridging analogies* where they fit securely. In keeping with the metaphor, scaffolding is a *temporary* structure to be removed when the building is complete. Perhaps a better metaphor (and still an engineering one) would be to provide *cantilevers*. These are *permanent* forms of support – as in the famous Scottish Forth (Rail) Bridge (A World-Heritage Site) – where three great cantilever structures support two relatively light central girders to form the arches. By analogy the massive supporting structures represent what the child already knows and the light supported structures represent future knowledge to be gained with the support of others. Thus the cantilevering metaphor embraces both Ausubel’s dictum concerning basing new knowledge on what the child already knows; and Vygotsky’s construct of learning from others within the ZPD. Educationally, Researchers and teachers should not/need not attempt to remove the old structures which remain in memory. They may be inhibited and suppressed but intact for cross-reference, according to the neuroscience – and so should be acknowledged as helpful to bridging between old learning and new learning^10^.

### Developmental aspects: the long view

Ausubel’s meaningful learning theory may be seen as a counter to Piaget’s theory of intellectual development (see Donaldson, 1978). Ausubel (1968) argued that a child should be taught according to what they already know, rather than according to “age” or “stage”. Bruner (1960) also refuted Piaget’s theory and argued: ‘We begin with the hypothesis that any subject can be taught effectively in some intellectually honest form to any child at any stage of development.‘ (p. 33). He advocated “*discovery learning*” whereby learners construct their own knowledge of the world. The construction of knowledge paradigm (constructivism) that evolved from the work of educational psychologists in the west was complemented by research and theories by Vygotsky (2012) that developed into social constructivism and socio-cultural learning. This work, and that of Luria, introduced the terminology: ZPD whereby children learn scientific concepts through interaction with adults and informed peers. The ZPD is closely related to the concept of scaffolding championed by Bruner (1960) which describes how children build on what they already know with help from others echoing Ausubel’s dictum.

The domain of children’s cosmologies has been fertile ground for research into developmental theories since the early work of Piaget (1929, 1930). Seminal work in this field was done by Nussbaum and Novak (1976) and Nussbaum (1979) who introduced the construct of *Earth notions* which classified children’s concepts on an ordinal scale from least to most scientific. Significantly, they concluded that their results were better informed by Ausubel’s theory rather than by that of Piaget. This was followed by cross-cultural research by Mali and Howe (1979, 1980) in Nepal; Klein (1982) in USA; Sneider and Pulos (1983) in USA; Brewer et al. (1987) in Samoa and USA; Vosniadou and Brewer (1992, 1994) in Greece and USA; Samarapungavan et al. (1996) in India; Diakidoy et al. (1997) with American-Indian (Lakota/Dakota) children in USA; and Vosniadou and Skopeliti (2017) in Greece into children’s mental models of the Earth and day/night.

Concurrently but independently, the current authors conducted a longitudinal ethnographic study of children’s cosmologies and associated fields from a variety of perspectives including cultural mediation, conceptual coherence, gravity thought experiments, switching between modes, imagery and interview methods (see Bryce & Blown, 2006; Blown & Bryce, 2010, 2013, 2017, 2020, 2022). These studies have been conducted against an evolving interpretation of children’s cognitive development including (a) for and against conceptual coherence versus knowledge-in-pieces (diSessa, 1988; Nobes et al., 2004); (b) the influence of cultural artifacts such as globes, maps and pre-made styrofoam models of the Earth in mediating mental models from a constructivist perspective (Vosniadou et al., 2005) and a socio-cultural view (Ivarsson et al., 2002; Schoultz et al., 2001); and (c) conceptual change vs. co-existing core systems of knowledge (Wiser & Carey, 1983; Carey & Spelke, 1994, 1996; Vosniadou, 2013). These studies highlighted the critical role of methodology in replicating results. Whereas Vosniadou et al. utilized the same open-ended questions in their studies with few cultural artifacts (pre-made Styrofoam models of the Earth); and reported a range of mental models from intuitive, through synthetic to scientific; Nobes et al. (2003); Ivarsson et al. (2002) and Schoultz et al. (2001) used forced choice questions with cultural artifacts (globes and maps) which inhibited intuitive and synthetic cosmologies (see Vosniadou et al., 2004).

From the perspectives of cognitive and developmental psychology, it is now recognized that the pioneering work of Piaget, Vygotsky and Bruner was too global in nature to be truly influential on school practice and, crucially, it underestimated the complexities of the developmental processes themselves. These deficiencies have been brought to light by the work of Donaldson (1978) on children’s intellectual development; Carey (1985, 2009) on conceptual development; Carey and Spelke (1994, 1996) on domain specific knowledge and conceptual change; Spelke (2000, 2005) on core knowledge and intrinsic aptitudes; Gelman (2003) on the essential characteristics of children; and Gopnik (1999) on the child as scientist. More recently Vosniadou et al. (2015) investigated executive function and conceptual change. They reported that “cognitive developmental research has shown that by the time systematic science instruction starts children have already constructed a naive physics, which is based on everyday experience and is very different from currently accepted science (Carey, 2009; Chi, 2008)”.

A common feature of this more recent research has been a move away from global, domain-general to child-centered, domain-specific learning and teaching to take greater account of individual differences. This trend is exemplified by the work of Demetriou, Mouyi et al. (2022) on cognitive competence, developmental difficulties, and school learning; and report that cognitive development occurs in cycles along several fronts that are considered to alter with changing developmental priorities which need to be recognized by teachers. In related work Demetriou, Spanoudis, Christou et al. (2022) drew on empirical data to show the developmental transitions taking place dominated by changes in attention control between 6 and 8 years, and by changes in working memory between 9 and 12 years. And most recently, Demetriou, Spanoudis, Greiff et al. (2022) reviewed research into how school performance relates to cognitive, self-awareness, language, and personality factors. The researchers outlined what they described as “the architecture of the mind, involving a general factor (g) that underlies distinct mental processes.” (p. 1). They reported that the focus of g changes with development, the emphasis switching from executive, reasoning and awareness functions to personality from childhood to adulthood. The authors concluded by advocating “a theory of educational priorities” (p. 1) featuring executive and awareness processes in preschool; information management at primary level; and reasoning, self-evaluation, and flexible knowledge building in secondary school.

## In conclusion

This review and analysis has allowed us to reflect critically on Ausubel’s work on meaningful learning. It has been written with considerable respect for the contribution he made to education and the challenges facing teachers on a daily basis. What we have argued stems largely from research conducted since he wrote about how learners assimilate new ideas from teachers and textbooks. At an important time, historically, he thereby also directed attention concerning the quality of possible learning in schools to the wider community – teacher educators, researchers and others concerned with curricular reform and educational developments.

At the heart of our critique is that while he characterized students’ previous knowledge so usefully, Ausubel (1960, 1963, 1968, 1978, 2000) paid insufficient attention to how prior learning was actually ascertained and with what surety. *Where* new ideas might fall on the meaningful-rote spectrum is shaped by the success of pedagogical exchanges which contend with the links being forged between old and new material. With hindsight, he was too focused on *static* conceptions of the constitution of existing ideas and their possible structure in the minds of the learner. He, and everyone else of his time, regarded the process of recollection as straightforwardly getting at what was there (or not). Researchers now know that the memory processes that are triggered by questions put to learners in the course of tuition are much more *dynamic* than was originally conceived; recollection is not the replicative act it was long considered to be. In Ausubel’s time, and prior to the advancements in neuroscience, memory was thought to be representational. Following Edelman’s work and that of several researchers in cognition, it is now considered that memory may be *non-representational* with a significantly creative dimension. Thus the act of recall is not a straightforward inspection of concepts. Its non-representational nature means that assumptions that are typically made about structures and associations concerning previously acquired knowledge are often not tenable. Putting it strongly, teachers probably have too much confidence in the postulated make-up of ideas which an individual possesses for everyday classroom topics. Furthermore, researchers can now say that the multimodal links that are forming in the minds of students during instructional exchanges are often not articulated or envisioned *unless* in-depth Socratic dialogue is sensitively pursued. Teachers must handle the *co-existence* of prior/naïve/everyday learning and the scientific expressions used in lessons. Multimodal thinking needs to be constantly encouraged. This means realizing that conceptual change is probably a process of conceptual *prioritizing, revision* and *selection* rather than *replacement*. Teaching requires one to be sensitive and positively reactive to alternative repertoires and ideas held by students. Doing so continually should encourage them to be open and responsive to what the teacher needs to know. As we stated earlier, both parties should know what they are mutually concerned to do during instruction. Education requires a reciprocity about learning.

It is tempting to speculate about the role of advance organizers in the light of recent research, in particular from a neuroscience perspective. Should researchers and teachers regard them as pathfinders or forms of concept mapping triggering effective neural pathways in preparation for future decisions and activity? The historical record of patchy findings about their effectiveness presumably underlines the considerable idiosyncrasies that exist among learners and how, so often, the Ausubelian approach fails to succeed – as perhaps so might any other strategy. Scaffolding, in the non-representational context, also needs to be carefully re-considered. It needs to be tackled with greater respect for the important role which early learning achieves. There is no case for ignoring how students previously conceived of closely relevant ideas; however there is merit in being explicit and open in classroom dialogue about the relationships between old and new. This is particularly so in the management of group work to prevent the spread of misconceptions/alternative frameworks amongst students.

## As a final point

In the (COVID-19) pandemic world, developed/developing countries are struggling to overcome the loss of so much schooling, the blight of ‘*blended (*or *hybrid) learning’*, excessive reliance upon on-line delivery of educational content, and difficulties with access to computers in school and home: Switzerland, Norway, Austria, 95%; Indonesia, 34% (Li & Lalani, 2020). Discriminating between scientific and non-scientific information on the Internet and social media is more important than ever, particularly given the degree of isolation from traditional sources of knowledge that has taken place. A recent exploration of the inter-related issues is contained in the Special Issue of *Science & Education, 31*, 5 devoted to “Trust in Science and Science Education” (see Erduran, 2022). Good teaching methods to recover the situation seem in short supply and, from the research perspective discussed here, revised Ausubelian perspectives on meaningful learning constitute a priority.

Ausubel advocated meaningful learning as a counter to the ages-and-stages paradigm in recognition of children’s capacity to learn at an earlier age than previously thought, provided that new learning was based on what children already knew. In order to optimize learning he created the construct of advance organizers which primed the child’s cognitive systems to be prepared to receive new information. His methodology was essentially child-centered with an emphasis on individual learning rather than whole class instruction. He disdained *discovery learning* in favor of *expository teaching* with the scientifically literate teacher as the primary source of knowledge. Over recent decades, research has certainly confirmed the importance of relevant basic (‘propositional’) knowledge as a requirement of any ‘guided discovery learning’. Klahr and Nigam (2004), for example, found not only that:

many more children learned from direct instruction than from discovery learning, but also that when asked to make broader, richer scientific judgments, the many children who learned about experimental design from direct instruction performed as well as those few children who discovered the method on their own. (Abstract)

And, to quote the findings from a large meta-analysis by Alfieri et al. (2011) of comparisons between unassisted *discovery learning* and explicit instruction, “unassisted discovery does not benefit learners, whereas feedback, worked examples, scaffolding, and elicited explanations do.” (p. 1).

The position in general is currently under threat as society moves increasingly towards alternative sources of knowledge such as the Internet. A situation that has been compounded by the COVID-19 pandemic resulting in high percentages of home schooling, less opportunity for one-to-one interaction with teachers, and limited access to books, libraries, and librarians. This is difficult for children and teachers in developed countries but is offset to some extent by access to online tutoring through computer programs such as Skype, Messenger, and Zoom video chat apps. However, children and teachers in developing countries have only limited access to such technology and are being severely disadvantaged the longer the pandemic continues. They may have no choice but to fall back on the traditional expository teaching of an experienced teacher in a classroom setting, taking whatever precautions they can (such as facemasks and hand-washing) to counter the COVID virus. Whatever their sources of knowledge about the world, children have to be taught to discriminate between reliable, truthful, scientific information and unreliable, untruthful, un-scientific misinformation. They should be encouraged to discuss their learning with their teachers, parents, librarians and more dependable peers in some form of collaborative learning.

## Footnotes

1 Namely Armita et al. (2014); Calderon (2015); Estrada (2015); Johnston (2008); Mitschek (2016); Saleh (2019); Sulleza (2015); and two graphic organizers: Athuraliya (2022); and Dawson (2022).

2 Novak (1993) traces the papers presented by Ausubel at earlier Faculty forums (at the Universities of Pennsylvania and Columbia) on ‘meaningful verbal learning’ and the “considerable difficulty [he had] in finding publication outlets in respectable journals of psychology or educational psychology” – such was the grip of behaviorism in the early 1960s, particularly in the USA.

3 We note the irony in Piaget being remembered for his ‘ages and stages’ (now regarded as outmoded), while Ausubel is forgotten as the founder of focusing teaching strategies upon individual differences in understanding, encapsulated by his construct of meaningful learning.

4 Piaget (1952) used the term *schema* to mean “a cohesive, repeatable action sequence possessing component actions that are tightly interconnected and governed by a core meaning” (p. 7). Schemas are ways of learning, organising knowledge and allowing people to systematize new perceptions.

5 Although Ausubel did not discuss Socratic dialogue his methodology would have been heavily dependent on such probing to determine children’s ideas. Conway (1979) helpfully stated: Socrates always sought to help his interlocutor discover the answers for himself, which is what is commonly understood by the ‘Socratic method’. In doing this Socrates always professed ignorance. The word for this in Greek is *eironeia* which means rather more than irony. Socrates pretends to not know the answer so that he can draw it out of the other person. (p. 12)

6 Hill and others have studied pedagogical content knowledge in the field of Mathematics. Writing about what they term Mathematical Knowledge for Teaching (MKT) in the course of their exploratory investigations with a number of teachers, they concluded that “there *is* a powerful relationship between what a teacher knows, how she knows it, and what she can do in the context of instruction” (Hill, Ball & Schilling, 2008). See also Hill, Blunk et al. (2008) and Adhikari (2020).

7 Where children are interviewed only once as in most cross-age research studies, teaching the child or arranging for the child to gain information from other scientifically minded adults is not a problem. But when children are being interviewed more than once by repeated measures in a longitudinal study then difficulties arise. Care must be taken to ensure that children in the survey group are not advantaged over the control group by receiving tuition from the researcher or others over and above that which the control groups are receiving. However, despite our best efforts to minimise the impact of the interview as a tutorial we found that survey groups gained enhanced concepts over and above those of the control groups (see Bryce & Blown, 2006; Blown & Bryce, 2010). These effects were detected by statistical analysis over time. They were not obvious in our earlier study (Bryce & Blown, 2006) where we reported little difference between survey and control groups (after an interval of five years); but became evident in our later study (Blown & Bryce, 2010) where the survey group had more advanced concepts than the control group (after ten years). (See Cromer, 1987, on linguistic pathways; Edelman, 2000, on re-entrant mapping).

8 Barsalou (2003) also uses the terms *simulator* and *simulation*. He stated (on page 521). that: “A concept is not a single abstracted representation for a category, but is instead a skill for constructing idiosyncratic representations tailored to the current needs of situated action. … [A] concept is a simulator that constructs an infinite set of specific simulations.”

9 The delays were associated with neural activity in regions handling error detection (such as inconsistencies in responses indicating cognitive conflict as concepts compete in a process where more plausible concepts are selected and less plausible are inhibited – a tug-of-war between everyday and scientific concepts).

10 Likewise, in Socratic dialogue used in research, researchers seek to find out what a child knows, both everyday and scientific, and direct the child to scientific sources (teachers and librarians) and sometimes (diverging from Socrates) teach rather than research. Sometimes we enhance children’s everyday repertoires as when discussing myths, legends, and folklore, but predominantly the teacher/researcher aims to impart scientific knowledge; e.g., explaining the scientific basis of lunar phases in relation to festivals in cultures using a lunar calendar.
